# Exploring genetic variants predisposing to diabetes mellitus and their association with indicators of socioeconomic status

**DOI:** 10.1186/1471-2458-14-609

**Published:** 2014-06-16

**Authors:** Börge Schmidt, Nico Dragano, André Scherag, Sonali Pechlivanis, Per Hoffmann, Markus M Nöthen, Raimund Erbel, Karl-Heinz Jöckel, Susanne Moebus

**Affiliations:** 1 Institute for Medical Informatics, Biometry and Epidemiology, University of Duisburg-Essen, Hufelandstraße 55, 45147 Essen, Germany; 2 Institute for Medical Sociology, Medical Faculty, University of Düsseldorf, Düsseldorf, Germany; 3 Clinical Epidemiology, Integrated Research and Treatment Center, Center for Sepsis Control and Care (CSCC), Jena University Hospital, Jena, Germany; 4 Department of Genomics, Life and Brain Center, University of Bonn, Bonn, Germany; 5 Institute of Human Genetics, University of Bonn, Bonn, Germany; 6 West-German Heart Center Essen, Department of Cardiology, University of Duisburg-Essen, Essen, Germany

**Keywords:** Health inequalities, Diabetes mellitus, Genetics of complex diseases

## Abstract

**Background:**

The relevance of disease-related genetic variants for the explanation of social inequalities in complex diseases is unclear and empirical analyses are largely missing. The aim of our study was to examine whether genetic variants predisposing to diabetes mellitus are associated with socioeconomic status in a population-based cohort.

**Methods:**

We genotyped 11 selected diabetes-related single nucleotide polymorphisms in 4655 participants (age 45-75 years) of the Heinz Nixdorf Recall study. Diabetes status was self-reported or defined by blood glucose levels. Education, income and paternal occupation were assessed as indicators of socioeconomic status. Multiple regression analyses were used to examine the association of socioeconomic status and diabetes by estimating sex-specific and age-adjusted prevalence ratios and their corresponding 95%-confidence intervals. To explore the relationship between individual single nucleotide polymorphisms and socioeconomic status sex- and age-adjusted odds ratios were computed. We adjusted the alpha-level for multiple testing of 11 single nucleotide polymorphisms using Bonferroni’s method (***α***_BF_ ~ 0.005). In addition, we explored the association of a genetic risk score with socioeconomic status.

**Results:**

Social inequalities in diabetes were observed for all indicators of socioeconomic status. However, there were no significant associations between individual diabetes-related risk alleles and socioeconomic status with odds ratios ranging from 0.87 to 1.23. Similarly, the genetic risk score analysis revealed no evidence for an association.

**Conclusions:**

Our data provide no evidence for an association between 11 diabetes-related risk alleles and different indicators of socioeconomic status in a population-based cohort, suggesting that the explored genetic variants do not contribute to health inequalities in diabetes.

## Background

Indicators of socioeconomic status (SES) are strongly related to health conditions with groups of low SES showing higher prevalence and incidence for almost all diseases [1-3]. Despite the evidence that differences in working and living conditions, health behaviors and psychosocial factors are important determinants underlying these health inequalities [4-6], twin and other types of family studies suggest a contribution of genetic factors due to selection effects [7-9]. However, on the molecular level the relevance of disease-related genetic variants for the explanation of social inequalities in health is still unclear and theoretical approaches as well as empirical analyses incorporating genetic data are largely missing. Learning more about the possible role of genetic factors in health inequalities is considered to further improve the understanding of population health in general and of health inequalities in particular.

If differences in genetic predisposition of a certain disease had an impact on health inequalities, it would be expected that the disease itself would have an influence on SES through intra- and intergenerational processes of social mobility, allowing for a higher frequency of disease-related risk alleles in lower SES groups [10]. This assumption is described by the hypothesis of direct health selection supposing that individuals with good health are more likely to move upward in SES than individuals with poor health and vice versa [11]. Incorporating genetic factors in the hypothesis of direct health selection would give disease-related risk alleles an impact on SES through a mediating effect of disease. However, direct health selection cannot be regarded as the main explanation for health inequalities because processes of social mobility are scarce in older ages when most diseases arise [11,12]. Particularly, if focusing on late onset diseases and measures of SES representing early life conditions or conditions across the life span (e.g., parental SES, education), reverse causation is unlikely.

With regard to common complex diseases it is supposed that next to environmental factors a large number of genetic variants contribute to disease etiology. In the recent past, disease-related risk alleles were primarily identified by genomewide-association studies (GWAS) following the *common disease – common variants* hypothesis [13]. These GWAS-based gene variants usually have small to modest individual effects [14], however strong relationships between common variants and complex traits would be necessary to gain a further prerequisite for supposed differences in allele frequencies between SES groups [15].

Hence, there is little reason to assume that differences in genetic predisposition to complex diseases play a role in health inequalities. To reconsider this argument by empirical analysis on the molecular level, the aim of this study was to examine whether there are associations between different SES indicators and genetic variants predisposing to diabetes mellitus as an example for a late onset complex disease. The inverse relationship between indicators of SES and diabetes is well reported across different populations [1,16-18]. In addition, a large number of genomic loci robustly associated with diabetes were identified through GWAS in the recent past [19-21]. To date, no studies have examined the association of SES and diabetes-related genetic variants to explore whether SES differences in genetic predisposition contribute to health inequalities in diabetes.

## Methods

### Study population

Data was used from the baseline examination of the Heinz Nixdorf Recall (Risk Factors, Evaluation of Coronary Calcium, and Lifestyle) Study, a prospective population-based cohort study. The rationale and design of the study were described elsewhere [22]. A random sample derived from mandatory citizen registries of three large cities (Bochum, Essen, Mülheim/Ruhr) in an urban region in the western part of Germany was used to recruit 4814 women and men aged 45–75 years. Baseline examination took place from 2000 to 2003 and the baseline response proportion was 55.8% [23]. Written informed consent was obtained from all participants. The study was approved by the institutional ethics committee of the University Hospital Essen and comprises extended quality management procedures, including a certification according to DIN ISO 9001:2000.

### Data assessment

Diabetes was defined as either of the following criteria: reported history of diabetes, taking glucose-lowering drugs, having fasting blood glucose levels of greater than 125 mg/dL, or having nonfasting glucose levels of 200 mg/dL or greater. Overall, 23 participants reported early disease onset indicating rather Type 1 than Type 2 diabetes. In analyses excluding these 23 participants virtually identical results were obtained as those presented in the following. To be consistent with a previous study using the same data [24] we decided to include the 23 participants in the analyses.

Based on literature research the following 11 single nucleotide polymorphisms (SNPs) related to 8 genetic loci (in parentheses) derived from GWAS for diabetes in European-origin populations were selected: rs4402960 (insulin-like growth factor-binding protein 2 [*IGF2BP2*]), rs1801282 (peroxisome proliferator-activated receptor gamma [*PPARG*]), rs7754840, rs7756992, rs10946398 (CDK5 regulatory subunit associated protein 1-like 1 [*CDKAL1*]), rs13266634 (solute carrier family 30 (zinc transporter), member 8 [*SLC30A8*]), rs10811661, rs564398 (cyclin-dependent kinase inhibitor 2A/2B [*CDKN2A/2B*]), rs7903146 (transcription factor 7-like2 [*TCF7L27*]), rs1111875 (hematopoietically-expressed homeobox [*HHEX*]) and rs8050136 (fat mass and obesity associated [*FTO*]). These SNPs include common variants with some of the highest effects on diabetes risk reported to date [19-21]. The literature research took place in January 2009 and was previously described in detail [24]. Genotyping was performed by matrix-assisted laser desorption ionization-time of flight mass spectrometry-based iPLEX Gold assay at the Department of Genomics, Life and Brain Center, Bonn, Germany.

Education, income and paternal occupation were collected as SES indicators by standardized interviews. Paternal occupation was classified referring to the International Standard Classification of Occupations (ISCO-88) [25] and categorized into four groups (unskilled employees/workers; qualified (skilled) employees/workers; technicians and associate professionals; managers and professionals).

Education was defined by combining school and vocational training as total years of formal education according to the International Standard Classification of Education [26] and categorized into three groups with the lowest educational group of 10 and less years (equivalent to a basic school degree with no vocational training), the medium educational group of 11 to 13 years (equivalent to upper secondary educational degrees or a combination of lower secondary education and vocational training) and the highest educational group of 14 and more years of education (equivalent to a vocational training including additional qualification or a university degree). In previous analyses of the same study population no further differentiation between university degrees and other types of higher education has been made because of the small number of diabetes cases in the respective group. This small sample size would have caused problems in conducting multivariate analyses.

Income was measured as the monthly household equivalent income calculated by dividing the total household net income by a weighting factor for each household member [27]. Income was included into analyses either as a continuous variable or divided into four groups using sex-specific quartiles.

### Statistical analyses

The analyses were conducted with 4655 participants who had information on diabetes status, indicators of SES and genetic data. Some observations on paternal occupation (n = 258), education (n = 14) and income (n = 296) were missing. As the SES indicators were analyzed separately, these participants were excluded only from the respective analyses. No correlations between missing SES measures and diabetes status were observed. All analyses were performed using the R statistical package version 2.14.0 [28] and PLINK (v1.07) for Windows [29].

First, log-binomial regression models were fitted to assess the association of SES indicators and diabetes status by estimating sex-specific and age-adjusted prevalence ratios (PR) and their corresponding 95%-confidence intervals (95%-CIs). Education, income and paternal occupation were entered separately as categorical predictors by coding dummy variables with the highest category as reference.

Second, logistic regression models were fitted to check the association of the GWAS-based SNP alleles to diabetes status. Therefore, sex- and age-adjusted odds ratios (OR) and 95%-CIs were estimated under a (log-)additive genetic model for each SNP, as suggested in previous studies [19,20]. In addition, a genetic risk score was developed by adding the risk alleles (0/1/2) of the diabetes-related SNPs for each participant to explore the association between the sum of risk alleles and diabetes status. For missing genotype information expected values were imputed based on the risk allele frequency of the respective SNP in the study population. SNP pruning for the genetic risk score was performed using pairwise linkage disequilibrium of *r*^*2*^ > 0.8 as cut off to account for correlated effects, resulting in the exclusion of rs1094639. The calculated effect size estimators are to be interpreted as average effects for one additional risk allele.

Third, for the primary research question the relationship between each SNP and SES was explored by computing sex- and age-adjusted ORs and 95%-CIs under a (log-)additive, dominant and recessive genetic model. Again, categorical education, income and paternal occupation were used separately as indicators of SES. For each SES category a binary outcome variable with the highest category as reference was entered in a logistic regression model. Income was also used as a continuous variable in a linear regression model to estimate standardized effect sizes and 95%-CI for each SNP. For this analysis income was log_e_-transformed to normalize the distribution. According to our knowledge, no association of the selected SNPs to SES has been demonstrated previously. Hence, it was decided to control the family-wise error rate of the primary research question at α = 0.05. We adjusted the alpha-level for multiple testing of 11 SNPs using Bonferroni’s method (α_BF_ ~ 0.005). In addition, the association between the genetic risk score and SES was explored using the SES indicators as outcome.

## Results

**Figure 1:**
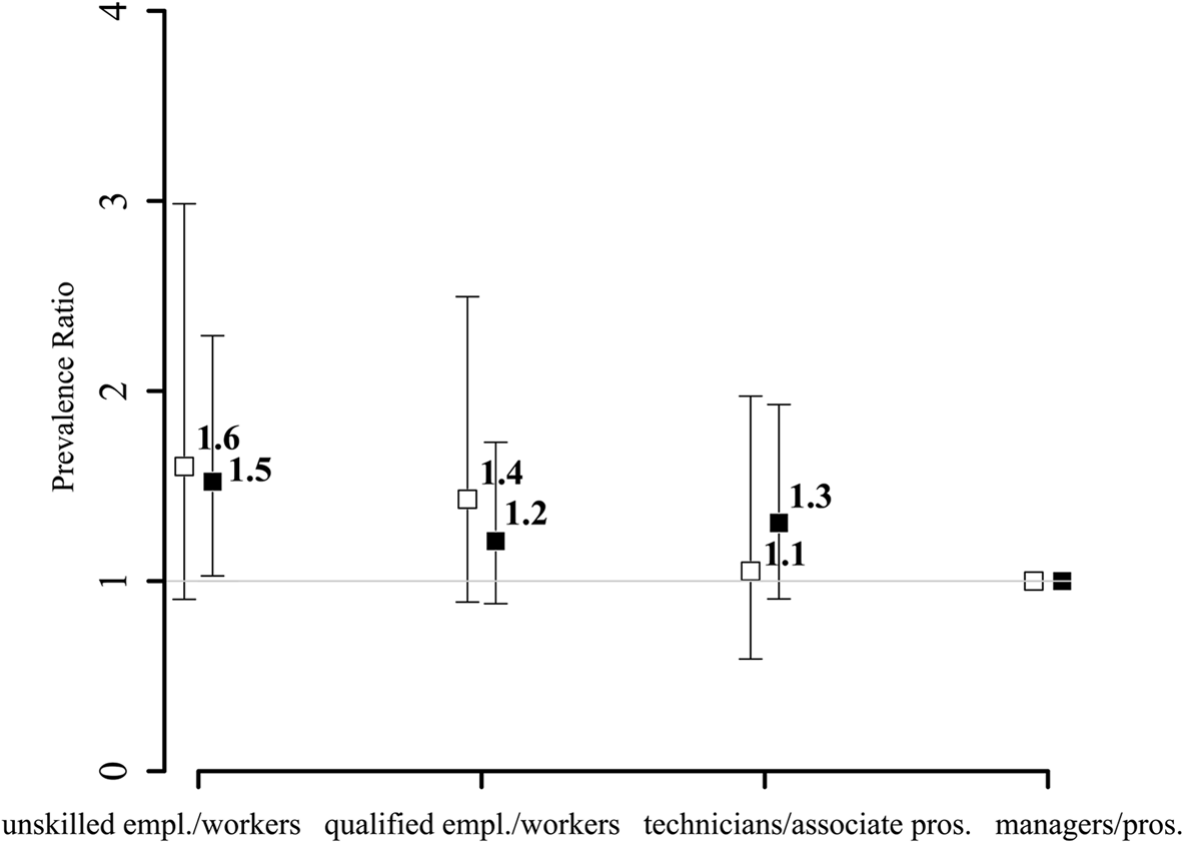
Paternal occupation and diabetes: age-adjusted prevalence ratios and 95% confidence intervals for the association of paternal occupation and diabetes for women (white) and men (black) (by groups; ‘managers and professionals’ as reference).

**Figure 2:**
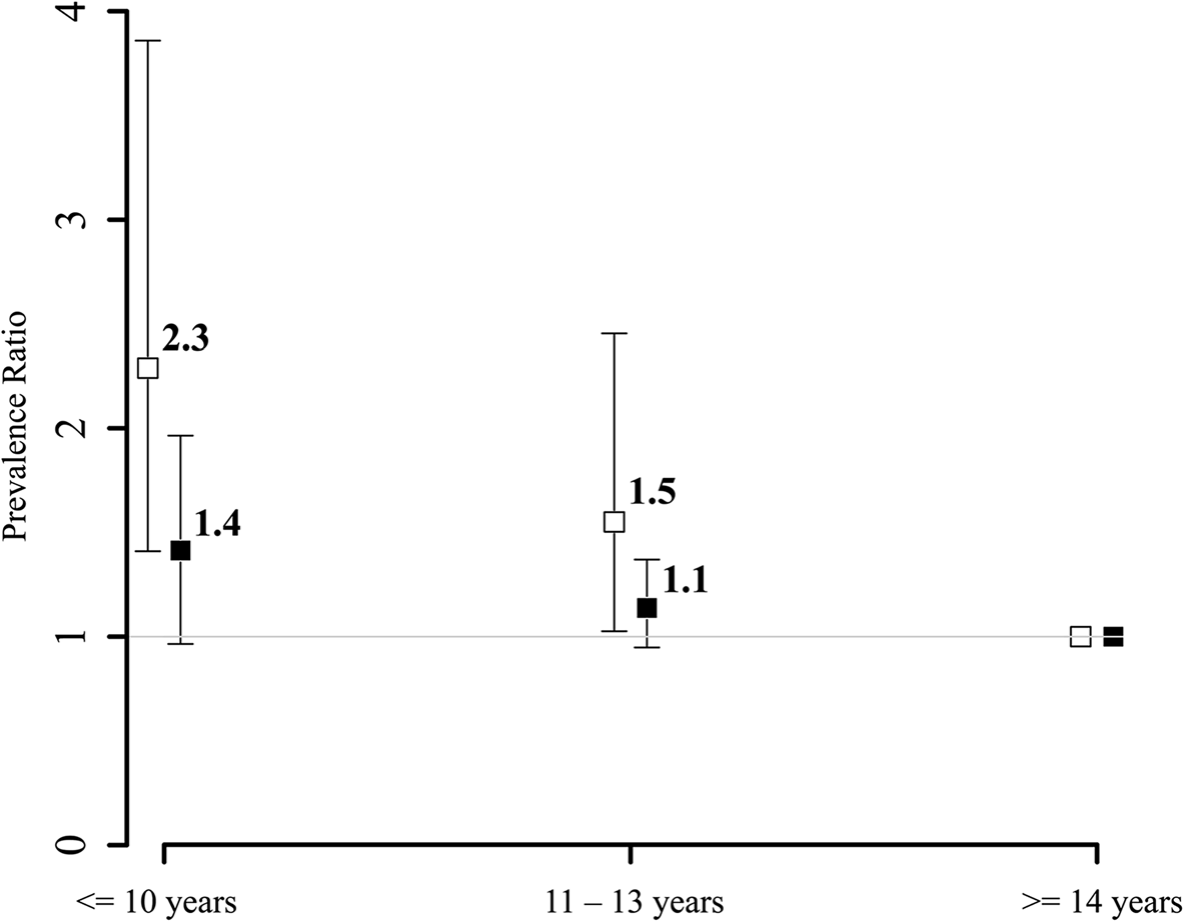
Education and diabetes: age-adjusted prevalence ratios and 95% confidence intervals for the association of education and diabetes for women (white) and men (black) (by groups; ‘> = 14 years of education’ as reference).

**Figure 3:**
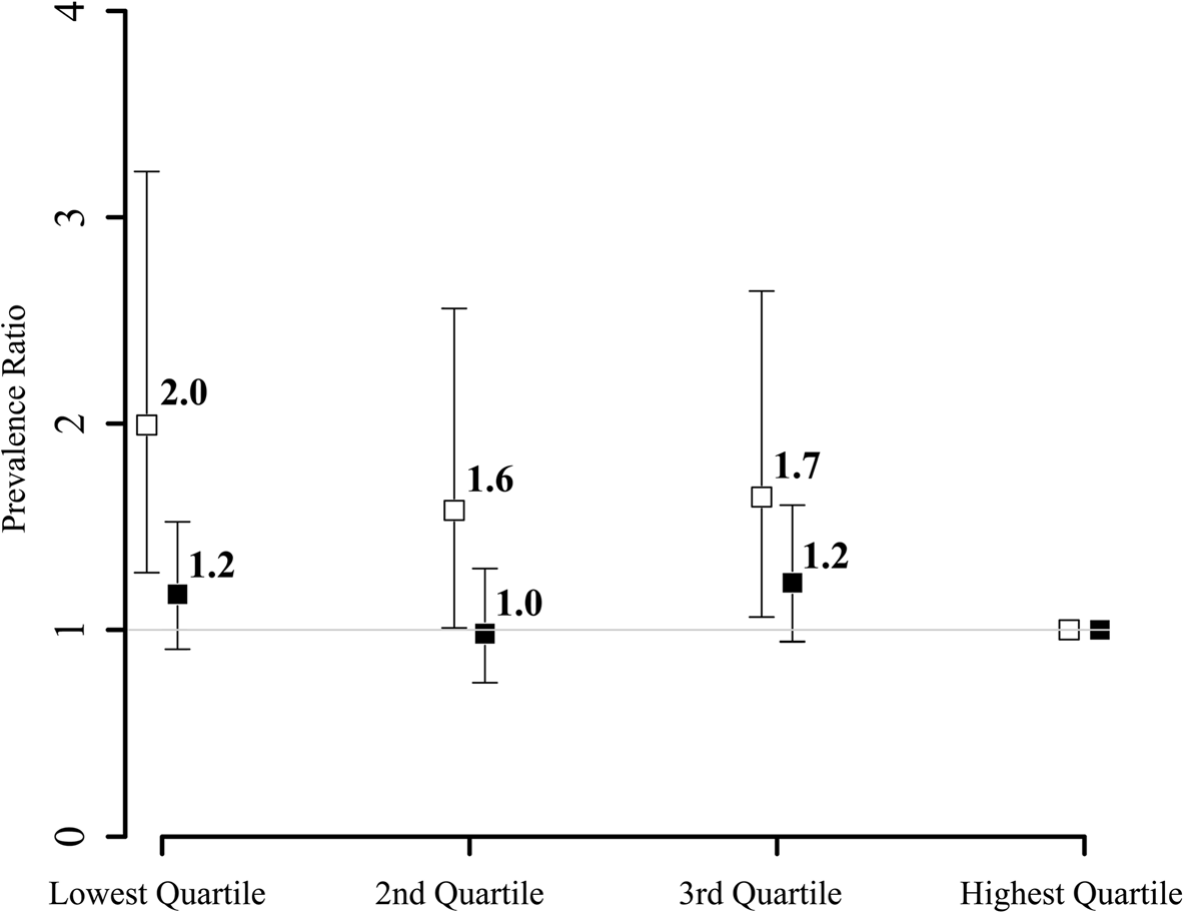
Income and diabetes: age-adjusted prevalence ratios and 95% confidence intervals for the association of income and diabetes for women (white) and men (black) (by sex-specific quartiles; highest quartile as reference).

Characteristics of the study population are shown in Table 1. Of the 4655 participants 13.6% (n = 634) had diabetes with women having a lower prevalence (9.8%) than men (17.4%). Differences between women and men in the distribution of two SES indicators were observed: Women reported less years of formal education and showed a lower median income.Inequalities in all SES indicators were related to diabetes status for both women and men (Figures 1, 2 and 3). The comparison of men in the lowest category of paternal occupation (unskilled employees/workers) to those in the highest category (managers and professionals) showed an age-adjusted PR of 1.5 (95%-CI: 1.0-2.3) for the occurrence of diabetes in the study population. The respective PR for women was 1.6 (95%-CI: 0.9-3.0). The analyses for education (≤10 years of education vs. ≥ 14 years; women: PR 2.3, 95%-CI: 1.4-3.9; men: PR 1.4, 95%-CI: 1.0-2.0) and income (lowest sex-specific quartile vs. highest sex-specific quartile; women: PR 2.0, 95%-CI: 1.3-3.2; men: PR 1.2, 95%-CI: 0.9-1.5) showed similar results. There are gender differences with women revealing stronger associations with diabetes status across all SES indicators.

**Table 1:** Characteristics of the study population

	**Female**	**Male**
**N**	2322	2333
**Age** (years)^a^	59.6 +/- 7.8	59.6 +/- 7.8
45-54^b^	725 (31.2%)	726 (31.1%)
55-64^b^	916 (39.4%)	917 (39.3%)
65-74^b^	681 (29.3%)	690 (29.6%)
**Diabetes mellitus**^b^	227 (9.8%)	407 (17.4%)
**Paternal occupation**^b^		
Unskilled employees/workers	270 (12.4%)	229 (10.3%)
Qualified (skilled) employees/workers	1283 (59.1%)	1363 (61.2%)
Technicians and associate professionals	392 (18.1%)	398 (17.9%)
Managers and professionals	226 (10.4%)	236 (10.6%)
**Education** (years of training)^b^		
≤10	411 (17.7%)	119 (5.1%)
11–13	1465 (63.2%)	1110 (47.8%)
≥14	443 (19.1%)	1093 (47.1%)
**Income** (EURO/month)^c^	1313 (937-1875)	1520 (1108-2073)
**Log**_**e**_**(income)**^a^	7.19 +/- 0.49	7.32 +/- 0.46

^a^mean (+/- standard deviation).

^b^number (%).

^c^median (interquartile range).

Table 2 shows the estimated ORs of the logistic regression models for the 11 selected SNPs and diabetes status. All effect size estimators are small to modest with ORs ranging from 1.01 to 1.28 for each risk allele. The SNPs rs4402960 (*IGF2BP2*), rs7756992 (*CDKAL1*), rs13266634 (*SLC30A8*), rs1111875 (*HHEX*), and rs7903146 (*TCF7L2*) showed statistically significant associations at a nominal α level of 0.05. For the genetic risk score a clear association with diabetes status could be observed (OR: 1.12, 95%-CI: 1.07-1.16, p: 5.95 × 10^-8^).

**Table 2:** Genetic association analyses for diabetes: odds ratios, 95% confidence intervals and p-values for the genetic association analyses for diabetes using SNPs (additive genetic model), sex and age in a logistic regression model (CHR, chromosome)

**CHR**	**Gene**	**SNP**	**Physical position**	**Supposed risk allele**	**Frequency in cohort**	**OR**	**(95% CI)**	***p***
3	*PPARG*	rs1801282	12393125	C	0.86	1.11	(0.92–1.33)	0.27
3	*IGF2BP2*	rs4402960	185511687	T	0.31	1.27	(1.12–1.44)	3.05 × 10^-4^
6	*CDKAL1*	rs10946398	20661034	C	0.33	1.07	(0.94–1.22)	0.28
6	*CDKAL1*	rs7754840	20661250	C	0.33	1.06	(0.93–1.20)	0.41
6	*CDKAL1*	rs7756992	20679709	G	0.29	1.16	(1.01–1.33)	0.04
8	*SLC30A8*	rs13266634	118184783	C	0.69	1.16	(1.01–1.32)	0.04
9	*CDKN2A/2B*	rs564398	22029547	A	0.58	1.09	(0.96–1.23)	0.21
9	*CDKN2A/2B*	rs10811661	22134094	T	0.83	1.01	(0.85–1.19)	0.96
10	*HHEX*	rs1111875	94462882	G	0.60	1.14	(1.01–1.29)	0.04
10	*TCF7L2*	rs7903146	114758349	T	0.27	1.28	(1.13–1.46)	1.67 × 10^-4^
16	*FTO*	rs8050136	53816275	A	0.41	1.08	(0.95–1.22)	0.23

The results for the logistic regression models for the 11 SNPs and paternal occupation under a (log-)additive genetic model are shown in Table 3. No statistically significant associations at α_BF_ ~ 0.005 were observed. The estimated ORs were small to modest ranging from 0.87 to 1.23 for each respective diabetes risk allele. The logistic regression models for the outcomes education (Table 4) and income (Table 5) revealed similar results with no statistically significant associations at α_BF_ ~ 0.005 and ORs ranging from 0.88 to 1.16 (education) and 0.87 to 1.14 (income). Under a dominant and recessive genetic model no deviant results to those under a (log-)additive model were obtained (results not shown). Furthermore, no statistically significant associations were observed for the analysis using log_e_-transformed income (results not shown). This was consistent with the observation for income as a categorized outcome.

**Table 3:** Genetic association analyses for paternal occupation: odds ratios, 95% confidence intervals and p-values for the genetic association analyses for paternal occupation (by groups; ‘managers and professionals’ as reference) using SNPs (additive genetic model), sex and age in a logistic regression model (CHR, chromosome)

					**Unskilled employees/workers**			**Qualified employees/workers**			**Technicians/associate pros.**		
**CHR**	**Gene**	**SNP**	**Physical position**	**Supposed risk allele**	**OR**	**(95% CI)**	***p***	**OR**	**(95% CI)**	***p***	**OR**	**(95% CI)**	***p***
3	*PPARG*	rs1801282	12393125	C	1.00	(0.77–1.30)	1.00	0.95	(0.77–1.17)	0.62	0.96	(0.76–1.22)	0.75
3	*IGF2BP2*	rs4402960	185511687	T	1.04	(0.86–1.26)	0.67	1.02	(0.87–1.18)	0.85	0.99	(0.83–1.18)	0.95
6	*CDKAL1*	rs10946398	20661034	C	1.01	(0.83–1.23)	0.94	1.15	(0.99–1.34)	0.08	1.17	(0.98–1.39)	0.09
6	*CDKAL1*	rs7754840	20661250	C	0.99	(0.82–1.21)	0.94	1.13	(0.97–1.32)	0.12	1.16	(0.97–1.39)	0.10
6	*CDKAL1*	rs7756992	20679709	G	1.06	(0.86–1.30)	0.59	1.13	(0.96–1.33)	0.15	1.23	(1.02–1.49)	0.03
8	*SLC30A8*	rs13266634	118184783	C	1.07	(0.88–1.31)	0.47	1.09	(0.94–1.26)	0.27	1.21	(1.01–1.46)	0.04
9	*CDKN2A/2B*	rs564398	22029547	A	0.95	(0.79–1.15)	0.63	0.94	(0.81–1.09)	0.39	0.94	(0.79–1.11)	0.46
9	*CDKN2A/2B*	rs10811661	22134094	T	0.98	(0.77–1.24)	0.85	1.10	(0.91–1.33)	0.33	0.94	(0.76–1.17)	0.59
10	*HHEX*	rs1111875	94462882	G	0.87	(0.72–1.05)	0.14	0.97	(0.84–1.12)	0.71	0.98	(0.83–1.16)	0.80
10	*TCF7L2*	rs7903146	114758349	T	0.89	(0.73–1.09)	0.26	0.92	(0.78–1.07)	0.28	1.01	(0.84–1.20)	0.95
16	*FTO*	rs8050136	53816275	A	1.09	(0.90–1.31)	0.38	1.05	(0.91–1.21)	0.53	1.00	(0.85–1.18)	1.00

**Table 4:** Genetic association analyses for education: odds ratios, 95% confidence intervals and p-values for the genetic association analyses for education (by groups; ‘> = 14 years of education’ as reference) using SNPs (additive genetic model), sex and age in a logistic regression model (CHR, chromosome)

					**<=10 years**			**11-13 years**		
**CHR**	**Gene**	**SNP**	**Physical position**	**Supposed risk allele**	**OR**	**(95% CI)**	***p***	**OR**	**(95% CI)**	***p***
3	*PPARG*	rs1801282	12393125	C	1.05	(0.82–1.34)	0.72	1.02	(0.89–1.17)	0.76
3	*IGF2BP2*	rs4402960	185511687	T	0.95	(0.80–1.13)	0.58	0.88	(0.80–0.98)	0.02
6	*CDKAL1*	rs10946398	20661034	C	1.08	(0.91–1.29)	0.37	1.01	(0.91–1.11)	0.89
6	*CDKAL1*	rs7754840	20661250	C	1.06	(0.89–1.27)	0.48	1.00	(0.91–1.11)	0.95
6	*CDKAL1*	rs7756992	20679709	G	1.07	(0.89–1.29)	0.45	1.00	(0.90–1.11)	0.95
8	*SLC30A8*	rs13266634	118184783	C	1.06	(0.89–1.27)	0.50	0.91	(0.82–1.01)	0.06
9	*CDKN2A/2B*	rs564398	22029547	A	1.04	(0.88–1.23)	0.66	1.00	(0.91–1.10)	0.99
9	*CDKN2A/2B*	rs10811661	22134094	T	1.16	(0.92–1.46)	0.21	1.13	(0.99–1.28)	0.07
10	*HHEX*	rs1111875	94462882	G	0.92	(0.78–1.08)	0.31	0.97	(0.88–1.07)	0.51
10	*TCF7L2*	rs7903146	114758349	T	1.04	(0.87–1.25)	0.64	0.98	(0.88–1.09)	0.71
16	*FTO*	rs8050136	53816275	A	0.96	(0.82–1.14)	0.65	0.95	(0.86–1.05)	0.30

**Table 5:** Genetic association analyses for income: odds ratios, 95% confidence intervals and p-values for the genetic association analyses for income (by sex-specific quartiles; highest quartile as reference) using SNPs (additive genetic model) and age in a logistic regression model (CHR, chromosome)

					**Lowest quartile**			**2nd quartile**			**3rd quartile**		
**CHR**	**Gene**	**SNP**	**Physical position**	**Supposed risk allele**	**OR**	**(95% CI)**	***p***	**OR**	**(95% CI)**	***p***	**OR**	**(95% CI)**	***p***
3	*PPARG*	rs1801282	12393125	C	1.14	(0.95–1.38)	0.15	0.98	(0.82–1.17)	0.82	1.02	(0.86–1.22)	0.80
3	*IGF2BP2*	rs4402960	185511687	T	1.00	(0.87–1.14)	0.97	0.96	(0.84–1.10)	0.59	0.96	(0.84–1.09)	0.50
6	*CDKAL1*	rs10946398	20661034	C	0.96	(0.84–1.09)	0.51	0.91	(0.80–1.04)	0.17	0.91	(0.80–1.03)	0.13
6	*CDKAL1*	rs7754840	20661250	C	0.96	(0.85–1.10)	0.59	0.92	(0.81–1.06)	0.24	0.90	(0.79–1.03)	0.11
6	*CDKAL1*	rs7756992	20679709	G	0.99	(0.86–1.14)	0.87	0.92	(0.80–1.06)	0.26	0.87	(0.75–1.00)	0.04
8	*SLC30A8*	rs13266634	118184783	C	0.99	(0.87–1.13)	0.88	0.98	(0.85–1.12)	0.75	0.98	(0.86–1.12)	0.73
9	*CDKN2A/2B*	rs564398	22029547	A	0.99	(0.87–1.13)	0.87	1.02	(0.90–1.17)	0.73	1.01	(0.89–1.15)	0.86
9	*CDKN2A/2B*	rs10811661	22134094	T	1.04	(0.88–1.24)	0.65	0.99	(0.83–1.17)	0.87	0.90	(0.76–1.06)	0.20
10	*HHEX*	rs1111875	94462882	G	0.94	(0.83–1.07)	0.35	0.86	(0.76–0.98)	0.02	0.94	(0.83–1.07)	0.35
10	*TCF7L2*	rs7903146	114758349	T	0.94	(0.81–1.07)	0.34	0.95	(0.83–1.09)	0.47	0.92	(0.80–1.05)	0.22
16	*FTO*	rs8050136	53816275	A	0.95	(0.84–1.08)	0.45	0.98	(0.87–1.11)	0.74	0.92	(0.82–1.05)	0.21

Table 6 shows the results using the genetic risk score in logistic regression models for all SES indicators. The estimated ORs are close to 1.0 and statistically significant results at a nominal α level of 0.05 were observed only for income comparing the 3^rd^ quartile with the highest quartile (OR 0.95, 95%-CI: 0.91-0.99).

**Table 6:** Genetic association analyses using a genetic risk score: odds ratios, 95% confidence intervals and p-values for the genetic association analyses for paternal occupation and education (both by groups; highest group as reference) using SNPs (genetic risk score), sex and age in a logistic regression model as well as for income (by sex-specific quartiles; highest quartile as reference) using SNPs (genetic risk score) and age in a logistic regression model

	**OR**	**(95% CI)**	***p***	**OR**	**(95% CI)**	***p***	**OR**	**(95% CI)**	***p***
**Paternal occupation**	unskilled employees/workers			qualified employees/workers			technicians/associate pros.		
	0.99	(0.94–1.05)	0.82	1.02	(0.98–1.07)	0.36	1.03	(0.98–1.09)	0.24
**Education**	<=10 years			11-13 years					
	1.02	(0.96–1.07)	0.52	0.98	(0.95–1.01)	0.15			
**Income**	lowest quartile			2nd quartile			3rd quartile		
	0.99	(0.95–1.03)	0.47	0.96	(0.92–1.00)	0.05	0.95	(0.91–0.99)	0.01

## Discussion

The presented data showed an association between all indicators of SES and diabetes status. Magnitude and trend of the associations across different SES groups are similar to those reported in the literature [1,17,18]. Stronger associations of SES differences in diabetes for women have been reported before as well [30]. With the selected SES indicators different but related aspects of social inequalities are measured representing different stages in the life course [18,31]. As associations for all of the explored SES indicators were found, our results give supporting evidence that health inequalities in diabetes are affected by diverse social conditions during different stages in life [18,32].

As expected, considering the given sample size and the relatively small number of diabetes cases in the study population, only 5 of the 11 selected diabetes-related SNPs (5 of the 8 genetic loci) were replicated with nominal statistically significant results for their association with diabetes status. However, all SNP alleles showed directionally consistent effects when compared with those reported in the literature [19-21].

The main finding of the study is the lack of evidence for the contribution of 11 selected diabetes-related SNPs representing 8 genetic loci to the observed health inequalities in diabetes. There were no statistically significant associations between the individual SNPs and SES indicators after conservatively controlling for multiple testing by the Bonferroni method. Even with an uncorrected level of significance (α = 0.05) the number of estimators with p < α does not exceed the number expected by chance.

In general, no clear inverse trend of the calculated ORs could be found across the different SES groups. There may be a few exceptions for markers related to *FTO* (for paternal occupation), *PPARG*, *CDKAL1* and *CDKN2A/2B* (for education). As the differences between the respective ORs are small and statistically significant results were missing, they have to be interpreted as random trends. Furthermore, we observed many ORs < 1.0 which we would not expect if the risk alleles of the selected SNPs generally have an impact on the observed health inequalities in diabetes. In addition, the highest ORs – which are only of small to modest magnitude – were not exclusively present for the lowest compared with the highest SES group. The results for using the genetic risk score in the analyses support the observations for the individual SNPs: The sum of selected diabetes risk alleles is not increasing with a decreasing in SES.

To our knowledge, this is the first study investigating social inequalities in diabetes and simultaneously exploring the impact of selected SNPs robustly associated with diabetes. There are just a few studies that have investigated supposed differences in risk allele frequencies to estimate their contribution to health inequalities with varying results [33,34]. Only Holzapfel et al. [34] analyzed the relationship between SES, body mass index (BMI) and the SNP rs9935401 within *FTO*, which is also associated with diabetes through its effect on BMI. They reported no association with education and income for rs9935401. This is in line with our observations for the *FTO* marker rs8050136, which is strongly correlated to rs9935401 (*r*^*2*^ = 1.0) within the HapMap CEU population.

Our study suggests that there is no contribution of diabetes-related SNPs to health inequalities in terms of differences in risk allele frequencies between SES groups. The question remains, how genetic factors could adequately be integrated in explanations of health inequalities. One possible approach is offered by the life course perspective [35-37], which describes the interplay of biological, environmental and social factors and their impact on health over the life course. Thus, the life course perspective is not tied to the assumption of a single causal direction as described by, e.g., the hypothesis of direct health selection. Following this approach, a more plausible picture of genetic risk factors interacting with environmental and social factors can be drawn to give consideration to the complex chain of risks that produces health inequalities.

The following limitations of the study need to be considered: First, due to the given sample size the statistical power to confirm the reported genetic associations with diabetes and to detect associations between the SNPs and the SES indicators – especially in the analyses with categorized SES indicators – is limited. To address this limitation and to increase the detection power we also used a genetic risk score for our analyses.

Second, over 66 genomic loci related to diabetes are already described which account for approximately 6% of variance in diabetes susceptibility suggesting that there still may be some unexplained genetic variance [38]. Thus, the present investigation of the relationship between diabetes-related genomic loci and SES indicators is far from being comprehensive. Given that the more recently discovered loci yield smaller effects on diabetes than the SNPs explored here, our analysis should be regarded as a first step to address the relationship of diabetes-related genomic loci with SES indicators.

Third, as we do not have information on diabetes status of earlier life stages it was not possible to check for the causal direction of the association between the SES indicators and diabetes. However, especially for education and paternal occupation reverse causation is unlikely as the former is a stable indicator of socioeconomic status across the life course and the latter of the participants’ childhood. For income, reverse causation cannot be ruled out.

Fourth, the validity of the SES indicators is restricted. For education and income this is due to their age dependency. The distribution of educational degrees varies by age groups with the higher groups showing lower variance and income generally declines in relation to retirement. Therefore, these indicators of SES may be more valid in younger age groups.

## Conclusions

Despite the mentioned limitations, our study confirms social inequalities in diabetes for different indicators of SES and provides no evidence that a selection of common genetic variants with the largest reported diabetes effects plays a role in the observed health inequalities. However, replication of our results, assessment of larger genetic marker panels and empirical analyses for other types of diseases are needed to further challenge the claims that differences in genetic predisposition could explain social inequalities in health.

## Competing interests

The authors declare that they have no competing interests.

## Authors’ contributions

KHJ, RE, SM and ND contributed to the conception, study design and data acquisition. BS conducted statistical analyses and contributed to the study design and interpretation of results. AS and SP contributed to statistical analyses and interpretation of results. MMN and PH carried out SNP genotyping. All authors contributed to manuscript preparation, and read and approved the final manuscript.

## Pre-publication history

The pre-publication history for this paper can be accessed here:

http://www.biomedcentral.com/1471-2458/14/609/prepub

## References

[B1] Dalstra JAA Kunst AE Borrell C Breeze E Cambois E Costa G Geurts JJ Lahelma E Van Oyen H Rasmussen NK Regidor E Spadea T Mackenbach JP Socioeconomic differences in the prevalence of common chronic diseases: an overview of eight European countries Int J Epidemiol 2005 34 316 326 10.1093/ije/dyh386 15737978

[B2] Siegrist J Marmot MG Social Inequalities in Health: New Evidence and Policy Implications 2006 Oxford: Oxford University Press

[B3] World Health Organization Closing the Gap in a Generation: Health Equity Through Action on the social Determinants of Health; Final Report 2008 Geneva: WHO

[B4] Aldabe B Anderson R Lyly-Yrjanainen M Parent-Thirion A Vermeylen G Kelleher CC Niedhammer I Contribution of material, occupational, and psychosocial factors in the explanation of social inequalities in health in 28 countries in Europe J Epidemiol Community Health 2011 65 1123 1131 10.1136/jech.2009.102517 20584725 PMC3678208

[B5] Matthews KA Gallo LC Psychological perspectives on pathways linking socioeconomic status and physical health Annu Rev Psychol 2011 62 501 530 10.1146/annurev.psych.031809.130711 20636127 PMC3121154

[B6] Skalická V van Lenthe F Bambra C Krokstad S Mackenbach J Material, psychosocial, behavioural and biomedical factors in the explanation of relative socio-economic inequalities in mortality: evidence from the HUNT study Int J Epidemiol 2009 38 1272 1284 10.1093/ije/dyp262 19661280

[B7] López-León S Chi Choy W Aulchenko YS Claes SJ Oostra BA Mackenbach JP van Duijn CM Janssens AC Genetic factors influence the clustering of depression among individuals with lower socioeconomic status PLoS ONE 2009 4 e5069 10.1371/journal.pone.0005069 19333388 PMC2659437

[B8] Osler M McGue M Christensen K Socioeconomic position and twins’ health: a life-course analysis of 1266 pairs of middle-aged Danish twins Int J Epidemiol 2006 36 77 83 17251245 10.1093/ije/dyl266

[B9] Silventoinen K Sarlio-Lähteenkorva S Koskenvuo M Lahelma E Kaprio J Effect of environmental and genetic factors on education-associated disparities in weight and weight gain: a study of Finnish adult twins Am J Clin Nutr 2004 80 815 822 15447885 10.1093/ajcn/80.4.815

[B10] Mackenbach JP Genetics and health inequalities: hypotheses and controversies J Epidemiol Community Health 2005 59 268 273 10.1136/jech.2004.026807 15767378 PMC1733045

[B11] Blane D Smith GD Bartley M Social selection: what does it contribute to social class differences in health? Sociol Health Illn 1993 15 1 15

[B12] Manor O Matthews S Power C Health selection: the role of inter- and intra-generational mobility on social inequalities in health Soc Sci Med 2003 57 2217 2227 10.1016/S0277-9536(03)00097-2 14512251

[B13] Frazer KA Murray SS Schork NJ Topol EJ Human genetic variation and its contribution to complex traits Nat Rev Genet 2009 10 241 251 19293820 10.1038/nrg2554

[B14] Hindorff LA Sethupathy P Junkins HA Ramos EM Mehta JP Collins FS Manolio TA Potential etiologic and functional implications of genome-wide association loci for human diseases and traits Proc Natl Acad Sci U S A 2009 106 9362 9367 10.1073/pnas.0903103106 19474294 PMC2687147

[B15] Holtzman NA Genetics and social class J Epidemiol Community Health 2002 56 529 535 10.1136/jech.56.7.529 12080161 PMC1732191

[B16] Agardh E Allebeck P Hallqvist J Moradi T Sidorchuk A Type 2 diabetes incidence and socio-economic position: a systematic review and meta-analysis Int J Epidemiol 2011 40 804 818 10.1093/ije/dyr029 21335614

[B17] Espelt A Borrell C Roskam AJ Rodríguez-Sanz M Stirbu I Dalmau-Bueno A Regidor E Bopp M Martikainen P Leinsalu M Artnik B Rychtarikova J Kalediene R Dzurova D Mackenbach J Kunst AE Socioeconomic inequalities in diabetes mellitus across Europe at the beginning of the 21st century Diabetologia 2008 51 1971 1979 10.1007/s00125-008-1146-1 18779946

[B18] Geyer S Hemström Ö Peter R Vagerö D Education, income, and occupational class cannot be used interchangeably in social epidemiology. Empirical evidence against a common practice J Epidemiol Community Health 2006 60 804 810 10.1136/jech.2005.041319 16905727 PMC2566032

[B19] Scott LJ Mohlke KL Bonnycastle LL Willer CJ Li Y Duren WL Erdos MR Stringham HM Chines PS Jackson AU Prokunina-Olsson L Ding CJ Swift AJ Narisu N Hu T Pruim R Xiao R Li XY Conneely KN Riebow NL Sprau AG Tong M White PP Hetrick KN Barnhart MW Bark CW Goldstein JL Watkins L Xiang F Saramies J A genome-wide association study of type 2 diabetes in Finns detects multiple susceptibility variants Science 2007 316 1341 1345 10.1126/science.1142382 17463248 PMC3214617

[B20] Sladek R Rocheleau G Rung J Dina C Shen L Serre D Boutin P Vincent D Belisle A Hadjadj S Balkau B Heude B Charpentier G Hudson TJ Montpetit A Pshezhetsky AV Prentki M Posner BI Balding DJ Meyre D Polychronakos C Froguel P A genome-wide association study identifies novel risk loci for type 2 diabetes Nature 2007 445 881 885 10.1038/nature05616 17293876

[B21] Voight BF Scott LJ Steinthorsdottir V Morris AP Dina C Welch RP Zeggini E Huth C Aulchenko YS Thorleifsson G McCulloch LJ Ferreira T Grallert H Amin N Wu G Willer CJ Raychaudhuri S McCarroll SA Langenberg C Hofmann OM Dupuis J Qi L Segrè AV van Hoek M Navarro P Ardlie K Balkau B Benediktsson R Bennett AJ Blagieva R Twelve type 2 diabetes susceptibility loci identified through large-scale association analysis Nat Genet 2010 42 579 589 10.1038/ng.609 20581827 PMC3080658

[B22] Schmermund A Möhlenkamp S Stang A Grönemeyer D Seibel R Hirche H Mann K Siffert W Lauterbach K Siegrist J Jöckel KH Erbel R Assessment of clinically silent atherosclerotic disease and established and novel risk factors for predicting myocardial infarction and cardiac death in healthy middle-aged subjects: rationale and design of the Heinz Nixdorf RECALL Study. Risk Factors, Evaluation of Coronary Calcium and Lifestyle Am Heart J 2002 144 212 218 10.1067/mhj.2002.123579 12177636

[B23] Stang A Moebus S Dragano N Möhlenkamp S Schmermund A Siegrist J Erbel R Jöckel KH Heinz Nixdorf Recall Study Investigation Group Baseline recruitment and analyses of nonresponse of the Heinz Nixdorf Recall Study: identifiability of phone numbers as the major determinant of response Eur J Epidemiol 2005 20 489 496 10.1007/s10654-005-5529-z 16121757

[B24] Pechlivanis S Scherag A Mühleisen TW Möhlenkamp S Horsthemke B Boes T Bröcker-Preuss M Mann K Erbel R Jöckel KH Nöthen MM Moebus S Heinz Nixdorf Recall Study Group Coronary artery calcification and its relationship to validated genetic variants for diabetes mellitus assessed in the Heinz Nixdorf recall cohort Arterioscler Thromb Vasc Biol 2010 30 1867 1872 10.1161/ATVBAHA.110.208496 20616309

[B25] International Labour Office International Standard Classification of Occupations: ISCO-88 1990 Geneva: International Labour Office

[B26] UNESCO International standard classification of education: ISCED 1997 [http://www.uis.unesco.org/Library/Documents/isced97-en.pdf]

[B27] Hagenaars A de Vos K Zaidi MA Poverty Statistics in the Late 1980s. Research Based on Micro-Data 1994 Luxembourg: Office for Official Publications of the European Communities

[B28] R Development Core Team R: A language and environment for statistical computing: R Foundation for Statistical Computing [http://www.R-project.org/]

[B29] Purcell S Neale B Todd-Brown K Thomas L Ferreira MA Bender D Maller J Sklar P de Bakker PI Daly MJ Sham PC PLINK: a tool set for whole-genome association and population-based linkage analyses Am J Hum Genet 2007 81 559 575 10.1086/519795 17701901 PMC1950838

[B30] Tang M Chen Y Krewski D Gender-related differences in the association between socioeconomic status and self-reported diabetes Int J Epidemiol 2003 32 381 35 10.1093/ije/dyg075 12777423

[B31] Galobardes B Shaw M Lawlor DA Lynch JW Davey Smith G Indicators of socioeconomic position (part 1) J Epidemiol Community Health 2006 60 7 12 10.1136/jech.2004.023531 16361448 PMC2465546

[B32] Tamayo T Christian H Rathmann W Impact of early psychosocial factors (childhood socioeconomic factors and adversities) on future risk of type 2 diabetes, metabolic disturbances and obesity: a systematic review BMC Public Health 2010 10 525 10.1186/1471-2458-10-525 20809937 PMC2940917

[B33] Gimeno D Ferrie JE Elovainio M Pulkki-Raback L Keltikangas-Jarvinen L Eklund C Hurme M Lehtimäki T Marniemi J Viikari JS Raitakari OT Kivimäki M When do social inequalities in C-reactive protein start? A life course perspective from conception to adulthood in the Cardiovascular Risk in Young Finns Study Int J Epidemiol 2008 37 290 298 10.1093/ije/dym244 18056120

[B34] Holzapfel C Grallert H Baumert J Thorand B Döring A Wichmann HE Hauner H Illig T Mielck A First investigation of two obesity-related loci (TMEM18, FTO) concerning their association with educational level as well as income: the MONICA/KORA study J Epidemiol Community Health 2011 65 174 176 10.1136/jech.2009.106492 20628085 PMC3251755

[B35] Kuh D Life course epidemiology J Epidemiol Community Health 2003 57 778 783 10.1136/jech.57.10.778 14573579 PMC1732305

[B36] Smith GD Health Inequalities: Lifecourse Approaches 2003 Bristol: Policy Press

[B37] Wadsworth ME Health inequalities in the life course perspective Soc Sci Med 1997 44 859 869 10.1016/S0277-9536(96)00187-6 9080567

[B38] Morris AP Voight BF Teslovich TM Ferreira T Segrè AV Steinthorsdottir V Strawbridge RJ Khan H Grallert H Mahajan A Prokopenko I Kang HM Dina C Esko T Fraser RM Kanoni S Kumar A Lagou V Langenberg C Luan J Lindgren CM Müller-Nurasyid M Pechlivanis S Rayner NW Scott LJ Wiltshire S Yengo L Kinnunen L Rossin EJ Raychaudhuri S Large-scale association analysis provides insights into the genetic architecture and pathophysiology of type 2 diabetes Nat Genet 2012 44 981 990 10.1038/ng.2383 22885922 PMC3442244

